# The positive externalities of migrant-based TB control strategy in a Chinese urban population with internal migration: a transmission-dynamic modeling study

**DOI:** 10.1186/s12916-021-01968-9

**Published:** 2021-04-20

**Authors:** Chongguang Yang, Jian Kang, Liping Lu, Xiaoqin Guo, Xin Shen, Ted Cohen, Nicolas A. Menzies

**Affiliations:** 1 Department of Epidemiology of Microbial Diseases, Yale School of Public Health, Yale University, New Haven, CT USA; 2 Department of Global Health and Population, Harvard T.H. Chan School of Public Health, Boston, MA USA; 3 Department of Tuberculosis Control, Songjiang Center for Disease Control and Prevention, Shanghai, China; 4 Department of Tuberculosis Control, Shanghai Municipal Center for Disease Control and Prevention, Shanghai, China

**Keywords:** Tuberculosis, Internal migration, Transmission-dynamic modeling, China, Public health policy

## Abstract

**Background:**

Large-scale rural-to-urban migration has changed the epidemiology of tuberculosis (TB) in large Chinese cities. We estimated the contribution of TB importation, reactivation of latent infection, and local transmission to new TB cases in Shanghai, and compared the potential impact of intervention options.

**Methods:**

We developed a transmission dynamic model of TB for Songjiang District, Shanghai, which has experienced high migration over the past 25 years. We calibrated the model to local demographic data, TB notifications, and molecular epidemiologic studies. We estimated epidemiological drivers as well as future outcomes of current TB policies and compared this base-case scenario with scenarios describing additional targeted interventions focusing on migrants or vulnerable residents.

**Results:**

The model captured key demographic and epidemiological features of TB among migrant and resident populations in Songjiang District, Shanghai. Between 2020 and 2035, we estimate that over 60% of TB cases will occur among migrants and that approximately 43% of these cases will result from recent infection. While TB incidence will decline under current policies, we estimate that additional interventions—including active screening and preventive treatment for migrants—could reduce TB incidence by an additional 20% by 2035.

**Conclusions:**

Migrant-focused TB interventions could produce meaningful health benefits for migrants, as well as for young residents who receive indirect protection as a result of reduced TB transmission in Shanghai. Further studies to measure cost-effectiveness are needed to evaluate the feasibility of these interventions in Shanghai and similar urban centers experiencing high migration volumes.

**Supplementary Information:**

The online version contains supplementary material available at 10.1186/s12916-021-01968-9.

## Background

China has an estimated 4.5 million prevalent tuberculosis (TB) cases, of which 80% arise among the rural population [1]. TB incidence in China has declined in recent decades, with national scale-up of directly observed treatment strategies and ongoing improvements in living standards. Currently, rural-to-urban migration is a major impediment for TB control in China. Most internal migrants are men who leave rural areas to join the urban wage economy, and there will be an estimated 240–260 million urban migrants by 2030. Due to China’s household registration system, most migrants typically face difficulties in becoming formal urban residents [2]. Consequently, they may not be entitled to subsidized housing or education, have poor access to social security and medical benefits, and often live and work in circumstances that promote *Mycobacterium tuberculosis* (*Mtb*) transmission and impede prompt diagnosis [3–5]. Studies have shown that urban migrants who develop TB have reduced access to TB care, and worse treatment outcomes than urban residents [6–8].

In China, identification of TB cases is primarily through passive case-finding, with symptomatic individuals voluntarily seeking diagnosis and treatment. High volume internal migration has been linked to elevated infectious disease incidence in Chinese cities, and challenges for disease control [6–8]. In recent years, rising TB case numbers associated with urban migration have been reported in many major cities—including Beijing, Shanghai, Shenzhen, Guangzhou, Wuhan and Hangzhou [4, 9–13]—yet there was no specific guideline for TB screening among urban migrants, and the underlying epidemiological mechanisms have not been completely described. The optimal response from urban TB control agencies will likely depend on the sources of new TB cases—whether elevated TB case notifications among urban migrants are due to reactivation of latent TB infection (LTBI) acquired before migration, transmission following migration, or prevalent TB among new arrivals.

In this study, we used a transmission-dynamic model to investigate the epidemiology of migration-linked TB in Shanghai. We conducted our study in Songjiang, a district of Shanghai in which the majority of the 2016 population were internal migrants. For Songjiang, previous genotypic analyses of clinical isolates have described transmission linkages between TB cases among residents and migrants [14]. Using these data, alongside detailed demographic and clinical data for 2000–2018, we estimated the contribution of different epidemiological mechanisms to TB incidence among migrants and residents and assessed the potential impact of new interventions focused on these population groups.

## Methods

### Study setting

Shanghai has experienced major rural-to-urban migration since the 1990s (Additional file 1: Figure S1) [15]. By 2016 the Shanghai population totaled 24 million, 41% of whom were internal migrants [16]. Songjiang has a population of 1.77 million, of which 62% are internal migrants, defined as people without a Shanghai household registration status through Chinese *hukou* system. Most originate from western and central China. In a previous study during 2006 to 2008, migrants had higher tuberculosis rates (38.9/100,000 population) compared to local residents (27.8/100,000 population) in Songjiang (Additional file 1: Figure S2) [6]. Since the 1990s, Songjiang has participated in a comprehensive TB surveillance and reporting system involving Songjiang District Centers for Diseases Control and Prevention, a TB-designated hospital, and community-based clinics. This network provides TB services including passive case finding, diagnosis, treatment, and follow-up. Data on TB cases, including demographic, epidemiological, and clinical information, is recorded in an electronic TB Information Management System.

Currently, Songjiang applies a referral system based on TB-like symptoms to prompt TB diagnosis and notification. Although there was no specific national guideline for TB control among internal migrants, since late 2004, an enhanced program was implemented in Shanghai to extend free TB services (reimbursement for diagnosis and anti-TB drugs) to the migrant population. Despite this expansion of standard TB care policy, there was no specific routine TB screening among migrants in Songjiang. Many local manufactory industries request a general pre-employment physical examination for both migrant and resident applicants, including chest X-ray. Residents aged 65 years and above can receive a voluntary annual physical examination, including a chest X-ray examination (not specific for TB screening).

### Study design and model structure

**Fig. 1:**
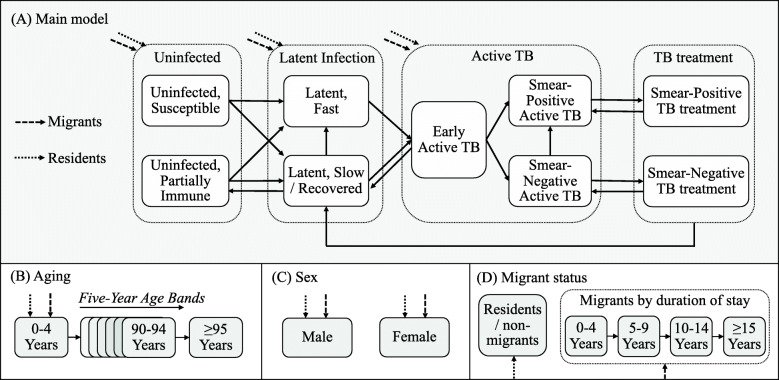
Schematic of TB simulation model. The schematic shows compartments within each dimension of the model: **a** Main model: core TB dimensions; **b** Age group dimension; **c** Gender dimension; **d** Risk group dimension by migration status. Arrows identify entries to the model and possible transitions between compartments. Solid arrows indicate state transitions, dotted arrows indicate model entry for resident individuals, and dashed arrows indicate model entry for internal migrant individuals. Migrants were divided into different status based on their duration of staying in Shanghai. Migrants exits due to death are not shown

We developed an age-structured transmission-dynamic model of TB epidemiology in Songjiang District, adapted from published models (Fig. 1) [17, 18]. The model stratifies the Songjiang population into four dimensions, with a core dimension representing TB transmission, natural history, diagnosis, and treatment (Fig. 1a and Fig. S4). Additional dimensions represented differences in population demography, TB burden and susceptibility, mixing patterns, and TB care access by age, sex, and migrant status (Fig. 1b–d). Migrant populations were stratified according to the duration of stay in Shanghai (Fig. 1d), with immigration and emigration modeled explicitly. Resident individuals entering Songjiang from other districts of Shanghai were not considered “migrants” for the analysis, as their epidemiological profile is expected to be similar to those of current residents and they held the same household registration status as the Songjiang local residents. We did not simulate transmission dynamics in rural areas and calibrated TB burden in incoming migrants to match observed disease patterns in Songjiang. The full model structure results from the crossing of the four dimensions and is represented with many distinct compartments and processes and the model code was detailed in Additional file 2.

### Data inputs

#### Demographic data

We extracted population data from the most recent National Census (2010), Shanghai 1% Population Sampling Survey (2015), and annual statistical yearbooks of Songjiang (2000–2018) [15, 16, 19]. These data included annual population by age and migrant status, distribution of the migrant population by reported years since entry and occupational category, birth rates, and mortality rates (Additional file 1:Table S1) [15]. Future migration estimates were based on Shanghai government population policy, which targets limited future population growth [20].

#### Observed TB cases and outcomes

From the TB surveillance system, we extracted TB case counts (available from 2000) stratified by age, gender, and migrant status. These data were used to build historical time-series of TB notification rates. We compiled additional evidence on TB care (including TB treatment discontinuation and treatment outcomes) from administrate data sources [6].

#### Transmission patterns

We used genotyping data that utilized an optimized MIRU-VNTR loci set (including the hypervariable loci) to estimate the extent of shared respiratory contacts among residents and migrants in Songjiang [14]. These data represent culture-confirmed TB patients reported in Songjiang from 2009 to 2015 and were used to estimate the probability that a clustered TB case in one of the two migrant strata (residents vs. migrants) would be due to transmission from an individual of the other group. Age-based mixing patterns were based on published contact matrices [21], and we allowed for increased respiratory contacts among 20–30-year-olds to account for elevated occupational exposure.

#### Natural history of latent infection and active disease

We specified parameters describing the probability of infection given *Mtb* exposure, rates of progression to TB for infected individuals, partial immunity of previously-exposed individuals, progression from early to late-stage disease, smear positivity (an indicator of infectiousness and mortality risk), infectiousness, TB-specific mortality and self-cure rates for individuals with active disease based on the published literature, and stratified by age and sex. Passive case detection was assumed to identify individuals with advanced TB for treatment, who could subsequently die, be cured, or return to untreated TB following treatment discontinuation or failure. We assumed that all diagnosed TB cases are reported, and that there was no treatment of LTBI under the base-case scenario. Apart from differential infection risks and TB treatment access, we assumed that natural history parameters would be the same between migrants and residents, conditional on age and sex. Tables S2-S3 (Additional file 1) provide parameter definitions, values, and data sources [6, 15, 22–32].

### Scenarios for future TB control options

The base-case scenario assumed continuation of current TB control activities, represented by current rates of treatment-seeking and current TB care outcomes. We compared this to hypothetical scenarios describing potential expansions in TB control. Table 1 describes model scenarios and operationalization [33–40]. In brief, scenarios one and two focus on improving TB prevention and care for elderly residents; scenarios three, four, and five target improvements in TB prevention and care for migrants. Scenario six represents the combination of migrant-focus interventions, and scenario seven represents the combination of all interventions included in other scenarios. We assumed any changes to current services would be introduced over 3 years, 2020–2022, then remain in place for the rest of the analysis period.

**Table 1:** Detailed model scenarios and operationalization

Scenarios	Approach	Model operationalization and parameters
Scenario 1. Improved TB case-finding in the elderly of residents (65+ years old)	Residents aged 65 years old and above currently receive a routine annual health check, which includes TB screening but with low sensitivity. This intervention would increase the sensitivity of this screen.	• Assumed to produce a rate of transition from early and late active disease states to treatment state. • Only applies to population 65 years and above among residents. • Transition rate calculated as rate of screening appointments * incremental improvement in sensitivity. • Rate of screening appointments = 1.0 • Incremental improvement in sensitivity = 10%
Scenario 2. Treatment of latent infection in the elderly (65+ years old)	At the annual health check-up for the elderly, residents screening TB negative would receive a TST, and those testing positive would receive prevent treatment (6H). Individuals would be screened once every 5 years.	• Only applies to population 65 years and above among residents. • For simplicity, assumed to exclude those with active TB and those on TB treatment. • Transition rate calculated as rate of screening appointments * TST sensitivity * treatment uptake probability * treatment completion probability. • Rate of screening appointments = 1.0 • TST sensitivity = 88% (95%CI 80–95) [33] • Treatment uptake probability = 35%(20–50%) [34–37] • Treatment completion probability = 70% (60–80%) [38] • Relative risk (RR) of developing active TB under prevent treatment (> 2 years) = 0.40 (0.30–0.50) [39]
Scenario 3. Pre-employment TB screening in migrants	A one-time active TB screening for migrants at time of first employment in the district.	• Assumed to produce a rate of transition from early and late active disease states to treatment state. • Only applies to migrants entering the model. • Applied as a probability of transition at the time of model entry. Probability calculated as screening sensitivity * diagnosis sensitivity * treatment initiation probability • Screening sensitivity = 90% (80–95%) for the late active disease (45%, for early status) [40] • Diagnosis sensitivity = 90% (80–95%) (smear test, liquid culture, and Xpert MTB/RIF) • Treatment initiation probability = 100%
Scenario 4. Pre-employment TB and LTBI screening in migrants	As in Scenario 3, plus LTBI screening and treatment for those screening negative for active TB	• Assumed to produce a rate of transition from latent fast and latent slow states to the partially immune state. • Applies to the same group as the scenario above. • For simplicity, assumed to exclude those with active TB and those on TB treatment. • Applied as a probability of transition at the time of model entry. Probability calculated as TST sensitivity * treatment uptake probability * treatment completion probability • TST sensitivity = 88% (80–95%) [33] • Treatment uptake probability = 35% (20–50%) [34–37] • Treatment completion probability = 70% (60–80%) [38] • Relative risk (RR) of developing active TB under prevent treatment (> 2 years) = 0.40, (0.30 to 0.50) [39]
Scenario 5. Routine active TB screening in factories	Regular active TB screening for individuals working in manufactories. Assumed screen algorithm: screening either for cough lasting for longer than 2 weeks, or screening for any symptom compatible with TB; Then chest radiography test.	• Assumed to produce a rate of transition from early and late active disease states to treatment state. • Applies to migrants work at the manufactory (62% of total migrant population, Fig. S3) • Transition rate calculated as rate of screening appointments * screening sensitivity * diagnosis sensitivity * treatment initiation probability • Rate of screening appointments = 1.0 • Screening sensitivity = 90% (80–95%) for the late active disease (45%, for early status) [40] • Diagnosis sensitivity = 90% (80–95%) (smear test, liquid culture, and Xpert MTB/RIF) • Treatment initiation probability = 100%

### Modeled outcomes

Outcomes included the number of effective *Mtb* transmissions by individuals with TB, the number of new infections resulting from transmission (these two outcomes respectively describing who transmits TB, and who is infected), incident TB cases, TB deaths, and life-years lost due to TB. We also decomposed new TB cases into those due to reactivation of established LTBI (infected > 2 years ago), recent transmission (infected < 2 years), and entry of individuals into Songjiang with prevalent disease. We reported outcomes per 100,000 of population, stratified by age and migrant status. We estimated outcomes until 2035 and reported results for 2020, 2025, and 2035.

### Statistics and operationalization

The model was programmed in R and C++ [41]. We used Bayesian evidence synthesis [42] to combine data and calibrate parameters to reproduce observed time-series of population and TB cases by age, sex, and migrant status. Calibration was implemented using Incremental Mixture Importance Sampling [43, 44], which produces a large number of epidemiological trajectories consistent with prior parameter distributions and calibration data (Additional file 1: Tables S4–5) [14]. We used the fitted model to simulate future outcomes under each analytic scenario. We calculated point estimates as the mean value across all simulated epidemiological trajectories and calculated 95% uncertainty intervals of the distribution of simulation results.

### Sensitivity analyses

In sensitivity analyses, we tested the impact of alternative assumptions of a 5% annual increase and 5% annual decrease in future migrant population size, which was held flat in the main analysis. Secondly, we tested alternative assumptions of 0% and 6% annual decline in LTBI and TB prevalence among future entering migrants, as compared to the 3% decline assumed in the main analysis. Thirdly, we assessed the impact of an extended 30-year time horizon (to 2050), as compared to 15 years used for the main analysis. Table S6 in the additional file 1 describes the additional parameters used for model sensitivity analysis [33–40].

## Results

### Comparison of model estimates to data on recent population and TB case trends

**Fig. 2:**
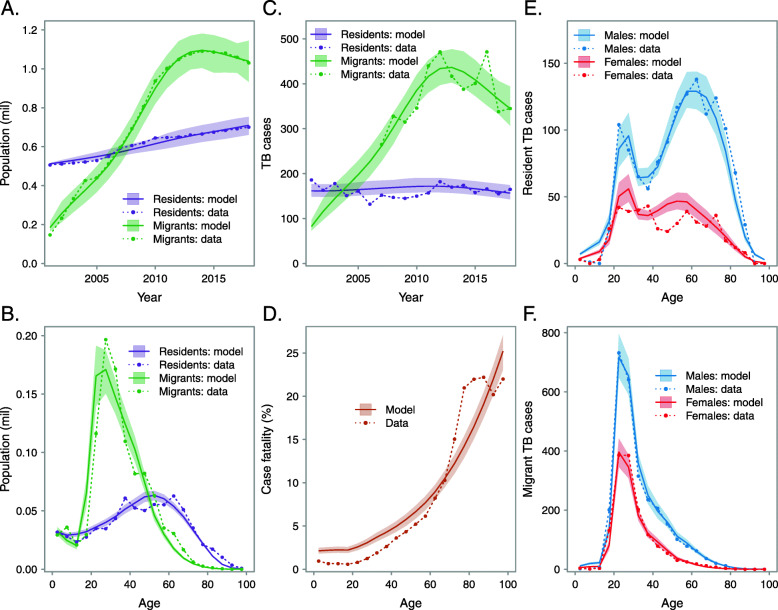
Comparison of model estimates and data describing population and TB outcomes by age and years: migrant and resident population (**a**, **b**), the annual number of diagnosed TB cases (**c**, **e**, and **f**), and case fatality rate (**d**). Model results are plotted for 1000 calibrated parameter sets to represent uncertainty in modeled results. The population size and age distribution data were abstracted from the national census and yearbook statistic records. TB case and facility data were drawn from the routine TB case reporting system

Recent population data show rapid population growth (from 0.1 to 1.1 million between 2000 and 2014, Fig. 2a), and a much younger age distribution (Fig. 2b) for migrants in Songjiang, relative to the resident population. Similar trends were observed in the distribution of TB notifications over time (Fig. 2c) and age (Fig. 2e, f), with migrant cases clustered in the 20–35 age group while resident cases demonstrated peaks at 20–35 and 60–80 years-old. By 2016 migrants represented approximately 60% of the overall population and 74% of all reported TB cases in Songjiang, had a higher notification rate (43.5 per 100,000) compared to that among resident (24.3 per 100,000), which were consistent with previous reports [6, 14]. Additional detail on these demographic and epidemiological trends is shown in Figs. S1–3 [6, 15, 16]. The model was able to reproduce these and other recent epidemiological data, including the TB case fatality estimates by age (Fig. 2d).

### Model estimates of TB epidemiology

**Fig. 3:**
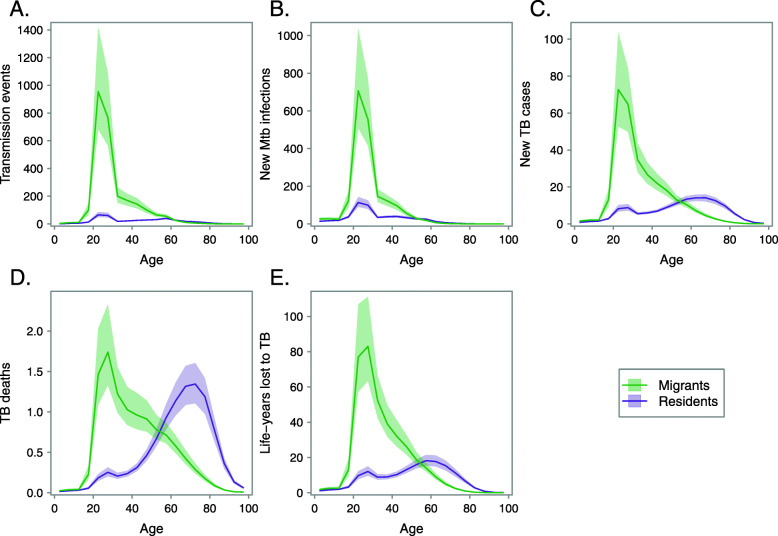
Model estimated key epidemiological outcomes in 2020 by age groups and migration status. Line represents the mean value and shaded areas represent 95% credible intervals. **a** The number of instances of *Mtb* transmission. **b** The number of new *Mtb* infections. **c** The number of new TB cases. **d** Deaths due to TB. **e** Life-years lost to TB

Figure 3 presents estimates for five key outcomes in 2020—effective *Mtb* transmissions by individuals with TB, new infections, incident TB cases, TB deaths, and years of life lost (YLL) from TB—stratified by age group and migrant status. Migrants in the 20–35-year-old age group represent a major fraction of each of these outcomes, reflecting their share of the population as well as elevated TB burden. Migrants were estimated to contribute 87.0% [95% uncertainty interval: 83.7, 89.9] of all instanced of TB transmission, and represent a smaller fraction (78.6% [74.4, 82.3]) of those newly infected, reflecting greater migrant-to-resident than resident-to-migrant transmission. Transmission from migrants was estimated to produce 91.7% [89.6, 93.9] of new infections among migrants and 69.9% [65.0, 73.6] of new infections among residents. The estimated fraction of all new infections caused by an individual of the other group (migrant to resident or resident to migrant) fitted well with the observed value (model 20.5% [18.8, 22.1], data 21.2%, supplementary materials). Residents represented an increased share of incident TB cases and 47.4% [42.3, 52.1] of TB deaths, reflecting elevated rates of reactivation TB and higher TB case fatality in the elderly resident population. Due to the younger average age, there was an estimated 35.2 [33.8, 36.7] YLL per TB death among migrants, compared to 16.5 [15.6, 17.4] YLL among residents. Migrants represented 70.2% [66.0, 74.4] of all life-years lost due to TB death.

**Fig. 4:**
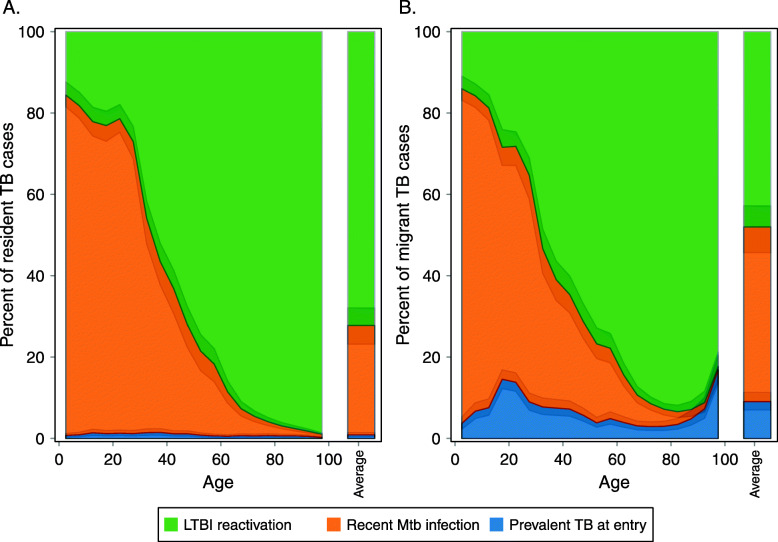
Model estimated fraction of new TB cases in 2020. The color represents the new TB cases due to LTBI reactivation (green), recent *Mtb* infection (orange), and prevalent TB among individuals migrating into Songjiang (blue), by age and migrant status (panel **a**, resident TB; panel **b**, migrant TB). The blue region in panel **a** represents residents who enter Songjiang from other districts in Shanghai. The polygon shows the distribution of new TB cases by age and the histogram presents the average of new TB cases from the three contributors. Faded color and border line represent the 95% uncertainty intervals of fraction

Figure 4 reports the fraction of new TB cases in 2020 due to LTBI reactivation, recent *Mtb* infection, and prevalent TB among individuals migrating into Songjiang, stratified by age and migrant status. For both migrants and residents, LTBI reactivation was estimated to represent the large majority of TB cases for individuals > 50 years old and was an important source of new TB cases, representing 72.2% [67.9, 76.8] of resident TB cases and 48.0% [42.9, 54.3] of migrant TB cases. Recent infection contributed 26.9% [23.2, 32.1] of resident TB cases and 42.9% [38.6, 45.8] of migrant TB cases, and was the primary cause of TB among individuals < 30 years old. Prevalent TB at entry to Songjiang contributed only 0.8% [0.5, 1.4] and 9.1% [7.0, 11.3] of TB cases among residents and migrants respectively. Overall, recent infection was estimated to cause 38.0% [33.3, 42.3] of incident TB cases overall.

### Epidemiological projections

**Fig. 5:**
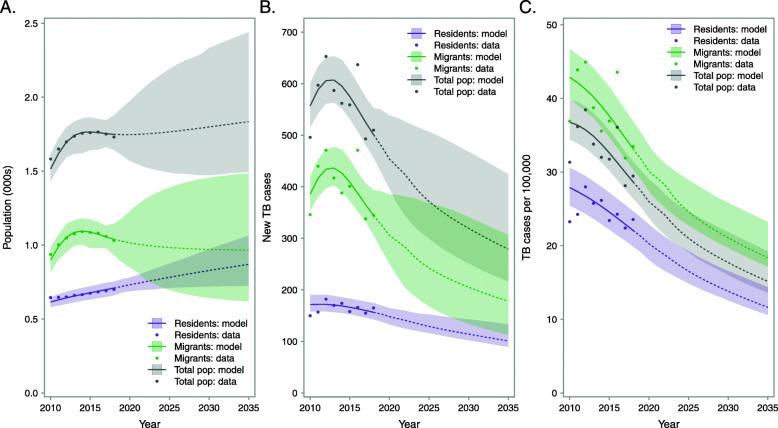
Projected trends in population size and TB incidence under base-case scenario in Songjiang, 2010–2035. Lines represent best estimate projection for a given population group. Dots represent the reported data. Shaded areas represent 95% credible intervals. **a** Migrant and resident population per thousand. **b** TB new cases. **c** Number of new TB cases per million

Figure 5 shows base-case projections of population and TB incidence. While the total population is projected to stabilize following rapid growth during 2000–2015 (Fig. 5a), TB cases are projected to decline. TB cases among the migrant population are projected to decline to 18.4 [17.1, 23.2] per 100,000 by 2035, a 49% [36–53] reduction from 2016. By 2035 resident TB cases are projected to decline to 11.6 [10.6, 14.4] per 100,000 (Fig. 5c), a 51% [39–55] reduction from 2016. These declines are consistent with recent TB case trends (Fig. 5, points). Migrants are projected to represent a relatively stable share of total TB cases, equivalent to 63% of total TB cases while representing 53% of the 2035 population.

Table 2 provides base-case projections for other TB outcomes. These outcomes are projected to follow similar trends to new TB cases, with TB transmission, new infections, TB deaths, and YLL due to TB all declining log-linearly over the projection period.

**Table 2:** Model estimated TB epidemiologic outcomes for the total population, migrants, and residents under base-case scenarios in 2015, 2020, 2025, and 2035

Population and year	Model outcomes, per 100 K									
	TB transmission events		New infection		New TB cases		Deaths due to TB		Life-years lost due to TB	
	Point estimate	95% CI	Point estimate	95% CI	Point estimate	95% CI	Point estimate	95% CI	Point estimate	95% CI
Residents										
2015	74	65, 85	96	83, 108	22.4	20.2, 25.1	1.64	1.36, 1.93	29.1	24.6, 35.0
2020	53	46, 61	71	61, 83	18.4	16.4, 21.0	1.33	1.09, 1.59	21.9	18.7, 26.4
2025	43	36, 50	62	52, 73	15.5	13.7, 17.8	1.15	0.94, 1.38	18.0	15.3, 21.6
2035	32	26, 38	51	40, 61	11.3	9.8, 13.2	0.85	0.69, 1.04	12.9	11.2, 15.4
Migrants										
2015	364	308, 430	255	216, 294	37.0	33.9, 40.6	1.37	1.14, 1.64	50.8	42.3, 59.0
2020	258	215, 314	191	164, 228	29.3	26.5, 32.8	1.06	0.89, 1.30	37.4	31.0, 44.9
2025	217	179, 269	169	140, 211	25.2	22.3, 28.4	0.91	0.77, 1.11	31.3	26.1, 37.6
2035	170	138, 218	140	112, 175	19.6	17.1, 22.9	0.7	0.58, 0.85	24.2	20.1, 29.5
Total										
2015	253	216, 294	194	165, 221	31.4	28.9, 34.1	1.47	1.27, 1.75	42.5	35.5, 49.3
2020	172	142, 214	141	120, 170	24.8	22.3, 28.1	1.17	1.01, 1.42	30.9	26.2, 36.3
2025	140	106, 185	121	94, 156	20.9	18.1, 23.8	1.01	0.86, 1.23	25.4	21.7, 30.8
2035	104	73, 141	98	71, 130	15.6	13.1, 18.6	0.77	0.66, 0.94	18.8	15.5, 23.6

### Alternative scenarios for interventions among residents and migrants

**Fig. 6:**
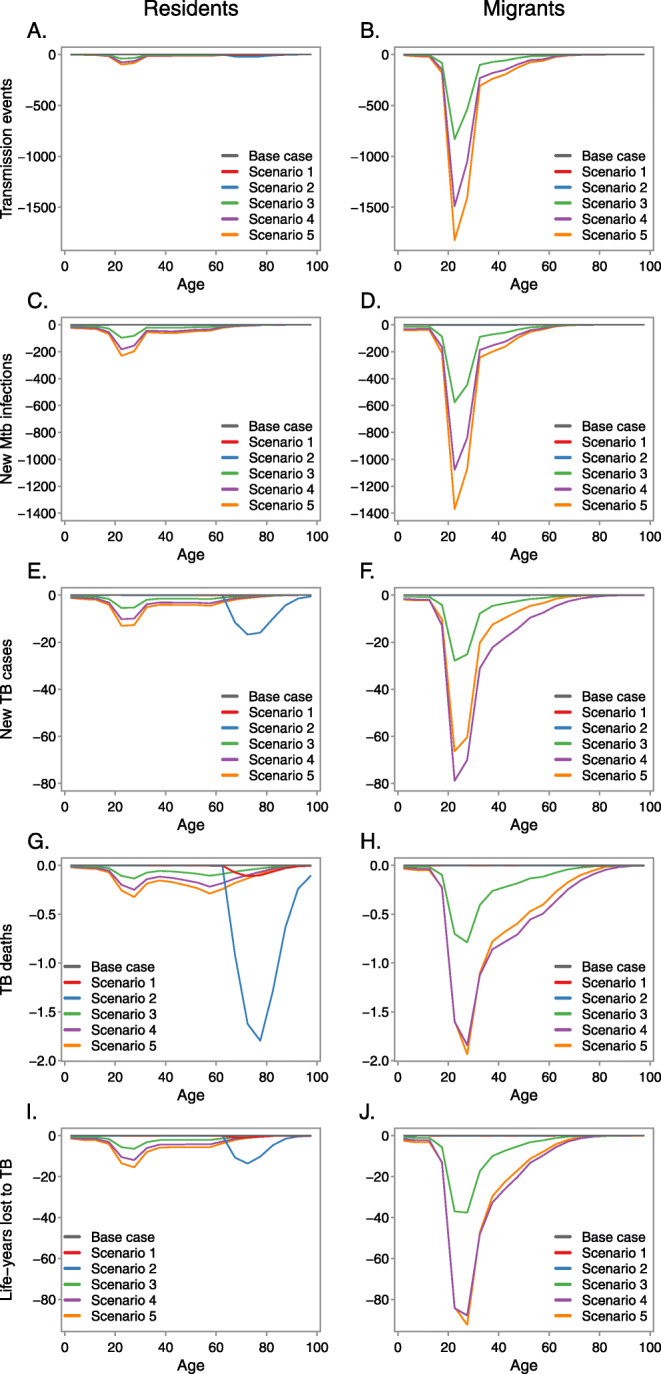
Model estimated incremental changes of key epidemiological outcomes for interventions scenarios 1–5 compared with the base-case from 2020 to 2035, stratified by age and migrant status. Resident outcomes (**a**, **c**, **e**, **g**, **i**) and migrant outcomes (**b**, **d**, **f**, **h**, **j**). Lines represent best estimate incremental changes for a given population group, scenario, and age group

Figure 6 shows incremental outcomes for the targeted interventions described by scenarios 1–5 compared with the base-case, summed over 2020–2035, and stratified by age and migrant status. Among these scenarios, interventions directed at the migrant population (particularly scenarios 4 and 5) are projected to have the greatest impact on most outcomes, with benefits concentrated in younger age groups consistent with the migrant age distribution. An exception to this pattern is for TB deaths, where scenario 2 is projected to have a major impact on TB deaths in the elderly resident population. In all cases, interventions targeted at migrants are projected to produce beneficial secondary effect for residents. In contrast, interventions targeted at residents are projected to have minimal impact on migrant TB outcomes.

Table 3 summarizes incremental reductions in TB outcomes for each scenario by 2035. In general, outcomes related to TB transmission (*Mtb* transmission, new *Mtb* infections) are more sensitive to intervention than other outcomes, with the combination of all interventions (scenario 7) projected to reduce these outcomes by 20–23% by 2035 compared to the base-case, while estimated reductions in other outcomes ranged from 10 to 12%.

**Table 3:**
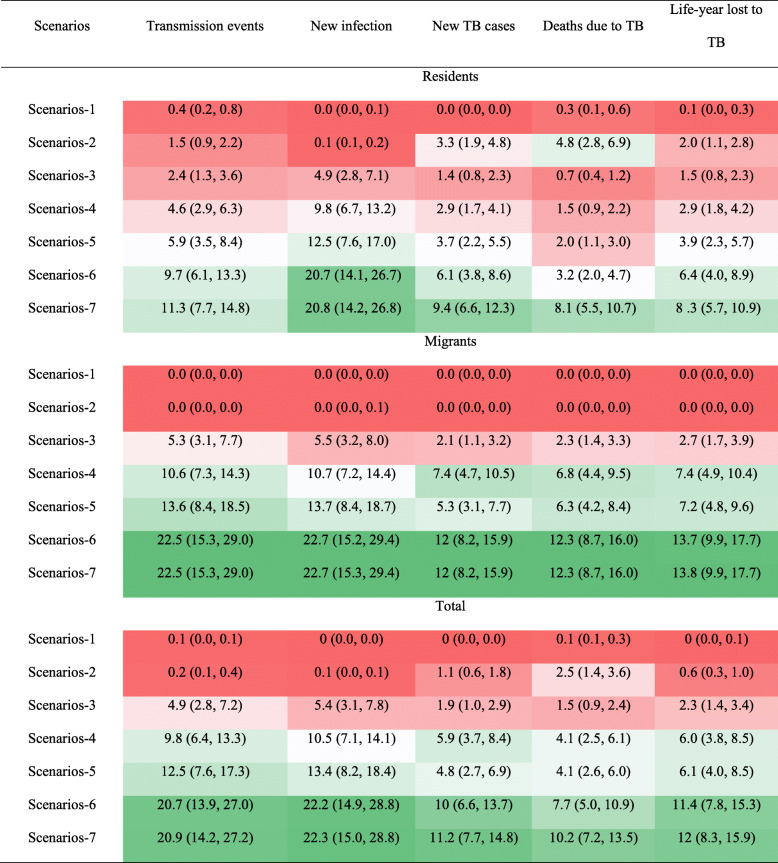
Model estimated cumulated incremental scenarios of TB epidemiologic outcomes by migration status as a percentage (95% CI) of reduction compared to their base-case value

Note: the color scale is specific to each outcome (i.e. each column) and consistent through the population groups. Scenarios 1–2, resident-focused interventions; scenarios 3–5, migrant-focused interventions; scenarios 6, combined migrant-focused interventions; scenario 7, all combined interventions

### Sensitivity analysis

Sensitivity analyses representing higher future migrant populations resulted in a greater effect of migrant-focused interventions relative to resident-focused interventions, and similar results were seen for sensitivity analyses representing slower declines in TB burden among future entering migrants. Even for sensitivity analyses representing faster future declines in migration volume and migrant TB burden, interventions focused on migrants still produced the greatest reductions in TB incidence and beneficial spillovers for resident populations. Results observed over a longer time horizon were consistent with the main analysis, with ongoing log-linear declines in TB burden when outcomes were projected to 2050. Over the long-term, intervention scenarios were shown to produce a step change in TB transmission, incidence and death rates, with this percentage difference with the base case increasing slowly over the extended simulation period. Detailed results for sensitivity analyses are shown in Fig. S5 and Table S7.

## Discussion

Over the past decades, there has been substantial growth of cities in China, driven by massive rural-to-urban internal migration [2]. This large-scale migration of young adults seeking economic opportunities has created opportunities for infectious disease transmission and has been associated with increases in TB case notifications. This poses new challenges for TB control in many urban areas, particularly in populous mega-cities like Shanghai, Beijing, Guangzhou, Wuhan, and Hangzhou [9–13]. In this study, we developed a mathematical model of TB that accounts for the demographic changes produced by high volume migration, and the differential risks of TB transmission, progression, and mortality across population groups. Using detailed local data on demography, case notifications, and molecular epidemiology, we calibrated the model to observed TB epidemiology in Shanghai, which is similar to other Chinese cities in terms of migration-linked population growth and concomitant increases in TB notifications [9–13]. We used this model to investigate the mechanisms underlying TB epidemiology in cities like Shanghai, and estimate the health impacts of alternative intervention strategies.

We found that large influxes of young migrants (adults predominantly 20–40 years old) has resulted in a high number of TB cases, new infections, and transmissions attributable to young adults in Shanghai. While the number of TB-related deaths is approximately equal between residents and migrants, because migrant cases are typically much younger than resident cases, the YLL due to TB is substantially larger for migrants. Our analysis suggests distinct mechanisms driving TB incidence in residents and migrants: for residents, approximately 70% of disease is due to reactivation of established latent infection, whereas for migrants, almost half of all TB cases are attributable to recent infection occurring after arrival in Shanghai. This was consistent with the findings from epidemiological observation studies in Shanghai [6, 14, 45]. We estimate that importation of prevalent disease among migrants represents a relatively small contribution to the TB burden in Shanghai. Importantly, our model also suggests very similar age-specific contributions of reactivation and recent infection. The difference in the relative importance of these mechanisms for the two population groups arises as a result of the fact that migrants are a much younger subgroup than residents, but within individual age strata, the relative contribution of each mechanism is similar.

Our analyses suggest that with the current TB control strategy, TB incidence in Shanghai will continue to decline slowly, and this slow downward trend is robust to realistic changes in migration volume. Our intervention scenarios were chosen to reflect current policy alternatives being discussed in China [46]. These included interventions focused on elderly residents, who are at high risk of developing TB (scenarios 1 and 2), and interventions focused on migrants (scenarios 3–6). Interventions focused on the elderly were estimated to have little incremental impact on the overall epidemic, but could produce sizable reductions in TB cases, deaths and YLL among the elderly. In contrast, pre-employment screening for disease (scenario 3) and treatment of latent *Mtb* infection (scenario 4) for recent migrants were projected to reduce TB cases and deaths in young migrants and produce secondary benefits for young residents with whom these migrants are likely to mix. Similar findings were noted in a population cohort study in the UK, that pre-screening of migrants could reduce later transmission risks [47]. If routine screening of factory workers can be brought to scale (scenario 5), we estimate that similar improvements in TB outcomes could be obtained as compared with pre-employment screening. Routine screening in factories may have advantages, as these factories are geographically concentrated in industrial zones and this might facilitate implementation of these interventions [13].

Several limitations in our study should be noted. Our projections are accompanied by uncertainty arising from incomplete knowledge of the natural history of TB, the current epidemiology of TB in Shanghai, and possible future changes in migration policy and disease burden. While we used detailed population, TB notification, and molecular epidemiological data from Shanghai to constrain model behavior to reproduce observed age- and migrant-status related TB epidemiology, uncertainty remains around serval model inputs, and as long-term model projections are difficult to validate, model design choices can bias future projections while still allowing good fit to current data. An influential model input was the MIRU-VNTR genotyping data, used to calibrate mixing assumptions. While the MIRU-VNTR loci set was optimized for local *Mtb* transmission in Songjiang, we cannot fully exclude risks of bias caused by low discrimination power of this method, or missed migrant cases due to emigration before disease detection. In addition, our analysis focused on the potential health benefits of interventions, yet other factors must be considered before adoption. Meanwhile, the current pandemic of COVID-19 could impact on the continuity of TB care and the future projection of TB transmission [48]. Further work is needed to investigate implementation strategies and costs for these interventions, to generate estimates of cost-effectiveness and examine feasibility.

## Conclusions

In summary, this analysis reveals that a substantial fraction of new TB cases in Shanghai are the result of local transmission in the city, driven by transmission involving young adults migrating for work. While this analysis was set in a relatively confined urban area—based on the rich data available in Songjiang for implementing the analysis—this epidemiological context is relevant for many large cities in China, with young adults migrating from rural areas with higher disease burden, and facing urban living conditions that allow the spread of respiratory pathogens. In this epidemiological context, we found that the introduction of interventions designed to interrupt local transmission related to migrants are likely to have greater population benefits than interventions directed toward elderly residents, the other main risk group for TB disease in this setting. This is related to both the greater transmission estimated for migrant groups and their large share of the population in this setting. While these new interventions could reduce TB incidence and death, we found that the magnitude of these benefits would be modest, with the most ambitious migrant-focused combination interventions achieving incremental reductions in TB incidence of approximately 10–15% by 2035. This suggests that current TB control strategies may be largely effective at achieving prompt treatment and limiting transmission, and greater innovation will be needed to meet high-level targets for TB control and elimination.

## Supplementary Information

**Additional file 1: Fig. S1.** Population by migrant status in Shanghai (1978–2016). **Fig. S2.** TB notification by migrant status and age group in Shanghai (2008–2016). **Fig. S3.** Occupations of migrants in Songjiang District, Shanghai, 2010. **Fig. S4.** Model structure and mechanisms. **Fig. S5.** Sensitivity of the estimated change of TB notification and incidence. **Table S1.** Birth and mortality rates of Songjiang District, Shanghai 2001–2018. **Table S2.** Model initial parameters – Demographics. **Table S3.** Model initial parameters – TB epidemiology and natural history. **Table S4.** Prior and posterior distributions for calibrated model parameters. **Table S5.** Distribution of cluster size of different cluster types, 2009–2015. **Table S6.** Additional parameters used for model sensitivity analysis. **Table S7.** Long-term horizon sensitivity analysis

**Additional file 2.** model data and code.

## Data Availability

All data generated or analyzed during this study are included in this published article and its supplementary information files.

## Abbreviations

TB: Tuberculosis
Mtb: *Mycobacterium tuberculosis*
LTBI: Latent tuberculosis infection
MIRU-VNTR: Mycobacterial interspersed repetitive unit-variable number tandem repeat
YLL: Years of life lost
