# Supplementary material for: The positive externalities of migrant-based TB control strategy in a Chinese urban population with internal migration: a transmission-dynamic modeling study
Source: BMC Med. 2021 Apr 20;19:95. doi: 10.1186/s12916-021-01968-9 (PMC8055441; doi:10.1186/s12916-021-01968-9)
Supplement: Supplementary file 1 — Additional file 1: Fig. S1. Population by migrant status in Shanghai (1978–2016). Fig. S2. TB notification by migrant status and age group in Shanghai (2008–2016). Fig. S3. Occupations of migrants in Songjiang District, Shanghai, 2010. Fig. S4. Model structure and mechanisms. Fig. S5. Sensitivity of the estimated change of TB notification and incidence. Table S1. Birth and mortality rates of Songjiang District, Shanghai 2001–2018. Table S2. Model initial parameters – Demographics. Table S3. Model initial parameters – TB epidemiology and natural history. Table S4. Prior and posterior distributions for calibrated model parameters. Table S5. Distribution of cluster size of different cluster types, 2009–2015. Table S6. Additional parameters used for model sensitivity analysis. Table S7. Long-term horizon sensitivity analysis [file 12916_2021_1968_MOESM1_ESM.docx]

**Additional file 1**

**The positive externalities of migrant-based TB control strategy in a Chinese urban population with internal migration: a transmission-dynamic modelling study**

Chongguang Yang, Jian Kang, Liping Lu, Xiaoqin Guo, Xin Shen, Ted Cohen, and Nicolas A Menzies

Contents

[S1. Study setting 2](#_Toc64390220)

[Figure S1. Population by migrant status in Shanghai (1978-2016) 2](#_Toc64390221)

[Figure S2. TB notification by migrant status and age group in Shanghai (2008-2016) 3](#_Toc64390222)

[Figure S3. Occupations of migrants in Songjiang District, Shanghai, 2010 3](#_Toc64390223)

[S2. Model representations and operationalization 4](#_Toc64390224)

[Figure S4. Model structure and mechanisms 5](#_Toc64390225)

[S3. Model Parameters and prior distributions 6](#_Toc64390226)

[Table S1. Birth and mortality rates of Songjiang District, Shanghai 2001-2018 6](#_Toc64390227)

[Table S2. Model initial parameters – Demographics. 6](#_Toc64390228)

[Table S3. Model initial parameters – TB epidemiology and natural history 7](#_Toc64390229)

[S4. Model calibration approach 8](#_Toc64390230)

[Table S4. Prior and posterior distributions for calibrated model parameters. 11](#_Toc64390231)

[Table S5. Distribution of cluster size of different cluster types 11](#_Toc64390232)

[S5. Scenario and Sensitivity analysis 12](#_Toc64390233)

[Table S6. Additional parameters used for model sensitivity analysis 14](#_Toc64390234)

[Figure S5. Sensitivity of the estimated change of TB notification and incidence 15](#_Toc64390235)

[Table S7. Long-term horizon sensitivity analysis 16](#_Toc64390236)

# S1. Study setting

Our study focuses on Songjiang—one of the sub-urban districts with a high influx of internal migration in Shanghai, China (Figure S1), covering an area of 605 km^2^. Roughly 1.10 million (62%) of Songjiang’s 1.77 million residents are internal migrants, whom we defined as people without a Shanghai household registration status through the Chinese hukou system. Most of the migrants in Shanghai have settled in seven suburban districts. Songjiang is one of the typical districts in terms of the fraction of the population that migrants comprise and their demographic characteristics patterns. Migrants in Songjiang mainly seek jobs in the manufactories located in the industrial zones (Figure S3). Internal migrants and their family members used not to share the same TB healthcare service as they can benefit from in their originally registered residence (mostly rural part of China). The influx of internal migration has contributed to the increased notification of TB in Shanghai (Figure S2). Since 2004, the Songjiang Center for Disease Control and Prevention (SCDC) implemented a new policy extending the TB service to all migrants.

## Figure S1. Population by migrant status in Shanghai (1978-2016)

* Shanghai Statistical Yearbook [15].

## Figure S2. TB notification by migrant status and age group in Shanghai (2008-2016)

* All form of pulmonary tuberculosis cases. Data source: Shanghai Center for Disease Control and Prevention (SCDC) [6].

## Figure S3. Occupations of migrants in Songjiang District, Shanghai, 2010

* The 2010 National Census in China [16].

# S2. Model structure and operationalization

# Our model subdivided TB natural history into a set of nine discrete health states, shown in Figure 1 and Figure S4. The TB states include: (1) Susceptible (i.e., never infected with TB), (2) Partially immune, Latent TB with (3) fast and (4) slow process to (5) Early active TB, Active TB with (6) smear-positive and (7) negative, and Treatment for (8) smear-positive and (9) negative TB. The LTBI states were stratified to allow variable rates of progression to active TB with time since infection (fast or slow progression). These states were further stratified according to age (20 strata), sex (2 strata), and migrant status (5 strata). We defined age strata based on the recorded national and Shanghai census data, with 5-year age bands from 0-4 years to 95+ years. For sex we defined male and female strata. For migrant status we defined on stratum for residents and four strata for migrants, subdivided according to their duration of residence in Shanghai (0-4 yrs, 5-9 yrs, 10-14 yrs, and 15+ yrs). Overall, this yields 1800 model compartments (9 TB states × 20 age strata × 2 sex strata × 5 migrant status strata).

The model was programmed in C++ using the Rcpp package, version 1.0.6 [41], and use the R version 4.0.1. The source code for this model is included as part of this supplement (Additional file 2-7: appendix model code). Model Figure S4 shows the main model of TB nature history, and transitions between model compartments. Tables S2 and S3 provide definitions and initial values for model parameters.

## Figure S4. Model structure and mechanisms*


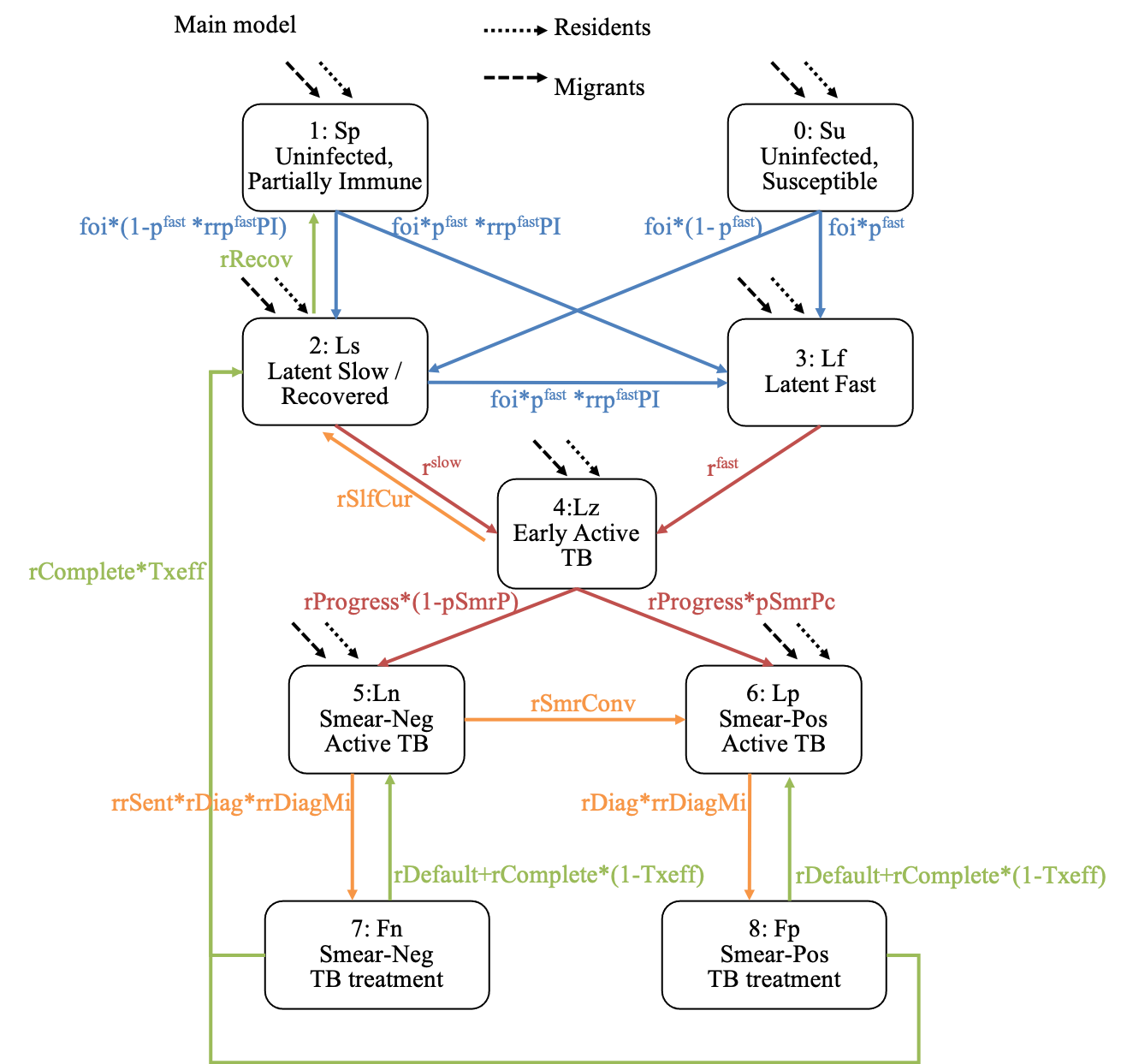


* Schematic shows parameterization for each model transition. Descriptions for each parameter are shown in Tables S2-S3. The force of infection (foi) at a given time *t* was calculated as $\lambda_{t}=\sum_{i} \frac{X_{it}}{N_{t}}\beta q_{i}$, where $X_{it}$ represents the population of each TB states, $N_{t}$ represents total population, $\beta$ represents the effective contact rate, and $q_{i}$ represents the relative infectiousness of each TB state.

# S3. Model parameterization and prior distributions

## Table S1. Birth and mortality rates of Songjiang District, Shanghai 2001-2018*.

| **Year** | **Birth rate, per 100K** | **Mortality rate, per 100K** |
| --- | --- | --- |
| 2001 | 644 | 687 |
| 2002 | 662 | 715 |
| 2003 | 658 | 745 |
| 2004 | 667 | 673 |
| 2005 | 701 | 695 |
| 2006 | 669 | 647 |
| 2007 | 749 | 632 |
| 2008 | 712 | 660 |
| 2009 | 727 | 652 |
| 2010 | 776 | 675 |
| 2011 | 755 | 652 |
| 2012 | 888 | 701 |
| 2013 | 784 | 694 |
| 2014 | 958 | 710 |
| 2015 | 847 | 739 |
| 2016 | 1083 | 706 |
| 2017 | 978 | 748 |
| 2018 | 978 | 748 |

* Songjiang District Yearbook [15]

## Table S2. Model parameters – Demographics.

| Name | Description | Value [Interval] | Source/Notes |
| --- | --- | --- | --- |
| *Birthst* | Birth rate (2001-2018) | Table S1 | Statistical Yearbook of Songjiang.[15] |
| *p_Male* | Proportion male among all births | 0.516 [0.506, 0.526] | Statistical Yearbook of Songjiang.[15] |
| *BgMort* | Mortality rate (2001-2018) | Table S1 | Statistical Yearbook of Songjiang.[15] |
| *Emg_rate* | Annual emigration rate | 0.2 [0.15, 0.3] | Shanghai migrants census in 1988, 1993 and 1997. [15] |
| *InitPrDur* | Distribution of initial migrant population as a function of time since entry | 0~4yrs 0.559  ≥ 5yrs 0.279  ≥ 10yrs 0.084  ≥ 15yrs 0.078 | National Census (2010).[6] |

## Table S3. Model parameters – TB epidemiology and care.

| Name | Description | Value [Interval]* | Source/Notes |
| --- | --- | --- | --- |
| ***TB transmission parameters*** | |  |  |
| *meanCR* | Average effective contact rate | 10 [7, 13] | [22]  Value calibrated to observed data |
| *rrFemCR* | Rate ratio of contact rates for female vs. male | 0.8 [0.4, 0.9] | Value calibrated to observed data |
| *rrMigCR* | Rate ratio of contact rates for migrants vs. residents | 1.0 [0.5, 1.5] | Value calibrated to observed data |
| *rrAgeCR20* | Rate ratio of contact rates for the 20-30-year-old population compared to other groups | 3.0 [1.0, 5.0] | Value calibrated to observed data |
| *rrInfect_Iz* | Infectiousness of early active TB relative to late active TB | 0.5 [0.3, 0.7] | Assumed. |
| *rrInfect_In* | Infectiousness of smear-negative TB relative to smear-positive TB | 0.23 [0.1, 0.4] | [23, 24] |
| *alpha* | Extent of mixing between migrant and resident groups. alpha = 1 indicates completely random; alpha = 0 indicates completely assortative within-group mixing. | 0.50 [0.20, 0.80] | Value calibrated to observed data |
| ***TB mortality parameters*** | |  |  |
| *mu_tb* | Population average TB mortality rate for smear positive TB | 0.1 [0.02, 0.2] | Tiemersma EW, et al.[25]  Value calibrated to reported case fatality. |
| *rrmuTb_age* | Rate ratio for the increase in TB mortality between 15-19 and 95-99 age groups | 10 [5, 20] | TB mortality rate assumed to be constant until the 15-19 age group, then increase log-linearly between 15-19 and 95-99 years of age. Value calibrated to reported case fatality estimates. |
| *rrmuTb_trt* | Mortality rate ratio for individuals on TB treatment vs. no treatment | 0.5 [0.2, 0.8] | Calibrated to reported data. |
| *rrmuTb_smrn* | Mortality rate ratio for smear-negative vs smear-positive TB | 0.5 [0.3, 0.7] | Tiemersma EW, et al.[25] |
| ***TB progression parameters*** | |  |  |
| *pfast* | Fraction of fast progression | 0.06 [0.003, 0.15] | Sutherland I.; Ferebee S., et al [26-27]  Value calibrated to observed data. |
| *RRpfastPI* | Risk ratio of fast progression for previously infected individuals | 0.21 [0.08, 0.40] | Andrews JR, et al. [28] |
| *rfast* | Rate of progression (fast) | 0.55 [0.19, 1.1] | Sutherland I.; Ferebee S., et al [26-27]  Value calibrated to observed data. |
| *rslow* | Rate of progression (slow) | 0.0007  [0.00004, 0.002] |  |
| *rrReactivate* | Rate ratio of TB progression in oldest age group | 1.0 [0.5, 2.5] | Calibrated to observed data |
| *rRecov* | Annual reduction in progression rate for individuals with distant infection | 0.03 [0.01, 0.06] | Sutherland I.; Ferebee S., et al [26-27] |
| *rSlfCur* | Rate of natural clearance of TB disease | 0.20 [0.10, 0.30] | Tiemersma EW, et al.[25] |
| *rProgress* | Rate of progression from early to late active TB | 3 [2.5, 3.5] | Assumed. |
| *pSmrP* | Fraction smear positive among active cases | 0.40 [0.25, 0.75] | Shen X, et al.[6]  Value calibrated to observed data. |
| *rSmrConv* | Rate of conversion from negative to positive | 0.15 [0.06, 0.29] | Hong Kong Chest S. [29] |
| ***TB diagnosis and care parameters*** | |  |  |
| *rDiag* | Rate of seeking treatment for late active TB | 3 [2, 4] | Value calibrated to observed data |
| *RRdiagMi* | Relative rate of seeking treatment for migrants vs. residents | 0.80 [0.70, 0.90] | Expert opinion |
| *dDetect* | Annual improvement in case-detection rate | 0.02 [0.001, 0.06] | Value calibrated to observed data |
| *rrSenst* | Relative sensitivity of diagnosis for smear negative vs. positive | 0.75 [0.70, 0.80] | Expert opinion |
| *rComplete* | Treatment completion rate | 2 | Annual rate requires to achieve an average 6-month treatment duration (fixed value). |
| *rDefault* | Treatment default rate | 0.18 [0.08, 0.31] | Shen X, et al. [6] |
| *Txeff* | Treatment cure probability | 0.96 [0.94, 0.98] | Menzies D, et al. [30]  Espinal MA, et al. [31]  Orenstein EW, et al. [32] |

* All rates parameters are given as annual rate (per person-year).

#

# S4. Model calibration

We calibrated the model to reduce the uncertainty around model inputs, and ensure the fitted model reproduced local demography and epidemiology, using reported information on the study population. We used a Bayesian approach to calibrate the model [42], implemented with Incremental Mixture Importance Sampling (IMIS) [43-44]. We constructed prior distributions for model parameters to reproduce the mean values and ranges shown in Tables S2 and S3. The functional form and parameterization of these priors is given in Table S4.

Table S4. Prior and posterior distributions for calibrated model parameters.

| **Parameters** | **Prior distributions** | | | | | **Posterior mean and 95% interval*** |
| --- | --- | --- | --- | --- | --- | --- |
|  | **Prior mean** | **Lower bound** | **Upper bound** | **Functional form** | **Hyper-parameters** |  |
| *pMale* | 0.516 | 0.506 | 0.526 | beta | *α* = 4951, *β* = 4641 | 0.52 [0.51, 0.53] |
| *Emg_rate* | 0.20 | 0.15 | 0.30 | gamma | *k* = 27.1, *θ* = 136 | 0.19 [0.17, 0.21] |
| *meanCR* | 10.0 | 7.0 | 13.0 | gamma | *k* = 42.5, *θ* =4.25 | 10.1 [9.4, 10.8] |
| *rrFemCR* | 0.80 | 0.40 | 0.90 | beta | *α* = 6.33, *β* = 1.58 | 0.91 [0.87, 0.95] |
| *rrMigCR* | 1.0 | 0.50 | 1.5 | gamma | *k* = 15.2, *θ* = 15.2 | 1.74 [1.62, 1.89] |
| *rrAgeCR20* | 3.0 | 1.0 | 5.0 | gamma | *k* = 6.66, *θ* = 3.32 | 2.04 [1.82, 2.33] |
| *rrInfect_Iz* | 0.50 | 0.30 | 0.70 | gamma | *k* = 23.8, *θ* = 47.7 | 0.61 [0.56, 0.66] |
| *rrInfect_In* | 0.23 | 0.10 | 0.40 | gamma | *k* = 8.86, *θ* = 38.5 | 0.18 [0.15, 0.21] |
| *alpha* | 0.50 | 0.20 | 0.80 | beta | *α* = 4.54, *β* = 4.54 | 0.79 [0.72, 0.85] |
| *muTb* | 0.10 | 0.02 | 0.20 | gamma | *k* = 4.57, *θ* = 45.7 | 0.19 [0.17, 0.21] |
| *rrmuTb_age* | 10.0 | 5.0 | 20.0 | gamma | *k* = 6.66, *θ* = 0.666 | 13.1 [11.3, 15.4] |
| *rrmuTb_trt* | 0.50 | 0.20 | 0.80 | beta | *α* = 4.54, *β* = 4.54 | 0.69 [0.61, 0.79] |
| *rrmuTb_smrn* | 0.50 | 0.30 | 0.70 | gamma | *k* = 23.8, *θ* = 47.7 | 0.50 [0.45, 0.55] |
| *pfast* | 0.06 | 0.003 | 0.15 | beta | *α* = 2.18, *β* = 34.2 | 0.081 [0.072, 0.091] |
| *RRpfastPI* | 0.21 | 0.08 | 0.40 | gamma | *k* = 7.36, *θ* = 35.0 | 0.14 [0.12, 0.18] |
| *rfast* | 1.0 | 0.5 | 2.0 | gamma | *k* = 15.2, *θ* = 15.2 | 1.02 [0.91, 1.18] |
| *rslow* | 0.0007 | 0.0001 | 0.002 | gamma | *k* = 1.92, *θ* = 2746 | 0.60 [0.49, 0.74] **10^-3^* |
| *rrReactivate* | 1.0 | 0.5 | 2.5 | gamma | *k* = 1.0, *θ* = 0.51 | 2.53 [2.33, 2.71] |
| *rRecov* | 0.03 | 0.01 | 0.06 | gamma | *k* = 8.47, *θ* = 282 | 0.026 [0.022, 0.032] |
| *rSlfCur* | 0.20 | 0.10 | 0.30 | gamma | *k* = 15.2, *θ* = 76.0 | 0.19 [0.16, 0.21] |
| *rProgress* | 3.0 | 2.5 | 3.5 | gamma | *k* = 138, *θ* = 46.0 | 2.84 [2.75, 2.94] |
| *pSmrP* | 0.40 | 0.25 | 0.75 | beta | *α* = 36.3, *β* = 54.4 | 0.39 [0.36, 0.43] |
| *rSmrConv* | 0.15 | 0.06 | 0.29 | gamma | *k* = 15.2, *θ* = 101 | 0.14 [0.13, 0.16] |
| *rDiag* | 3.0 | 2.0 | 4.0 | gamma | *k* = 34.4, *θ* = 11.5 | 3.06 [2.78, 3.35] |
| *rrDiagMi* | 0.50 | 0.35 | 0.65 | beta | *α* = 16.4, *β* = 5.45 | 0.79 [0.75, 0.83] |
| *dDetect* | 0.03 | 0.02 | 0.05 | gamma | *k* = 34.4, *θ* = 459 | 0.054 [0.049, 0.058] |
| *rrSenst* | 0.75 | 0.70 | 0.80 | beta | *α* = 864, *β* = 1152 | 0.75 [0.70, 0.80] |
| *rDefault* | 0.18 | 0.08 | 0.31 | gamma | *k* = 73.9, *θ* = 168 | 0.42 [0.40, 0.45] |
| *Txeff* | 0.96 | 0.94 | 0.98 | beta | *α* = 83.5, *β* = 3.48 | 0.96 [0.94, 0.98] |

* Intervals represent equal-tailed 95% posterior intervals.

We calibrated the model to data from two primary sources. First, we used demographic data provided by the National Census publications to obtain the estimates of migration rate, population size, sex, and age structure by migrant status (i.e. local resident or migrant). Second, routine surveillance data of Songjiang district from the China TB Information Management System (TBIMS) were used to derive estimates of the TB notification rate by sex, age group, and migrant status. The calibrated data source was detailed in the appendix data and code. In addition, we calibrated the model to data on the transmission linkages between migrants and residents based on a prospective molecular epidemiology study in Songjiang district conducted between Jan. 2009 and Dec. 2015 [14]. Genotypic clustering was assumed to be a proxy index for transmission relatedness of cases within the same cluster. We estimated the transmission probability of clustered individuals from migrant or residents based on the cluster type and the proportion of patients being migrant and residents in the cluster: 1). Force of infection by migrant (*p_migrant_contact*): for a specific cluster, the possibility of one case in this cluster infected by migrant equals the sum of cases being migrant in this cluster / the cluster size. 2). Force of infection by resident (*p_resident_contact*): for a specific cluster, the possibility of one case in this cluster infected by resident equals the sum of cases being resident in this cluster / the cluster size. We calibrated our model to the fraction of clustered cases infected by an individual of the other type (i.e., migrant to resident or resident to a migrant). The model estimated a value of 0.205, which fits well with the reported data (0.212). The genotypic data (n=488) of VNTR clustering analysis of the culture-positive TB cases in Songjiang is shown in Table S5. Parameter posterior mean values and 95% intervals summarizing the calibration are shown in Table S4.

Table S5. Distribution of cluster size of different cluster types (genotypic data obtained between 2009 and 2015) [14]

| Cluster size | Clustered patients among mixed clusters | | | Clustered patients among resident or migrant only clusters | |
| --- | --- | --- | --- | --- | --- |
|  | Total | Resident | Migrant | Resident | Migrant |
| 2 | 64 | 32 | 32 | 42 | 156 |
| 3 | 42 | 18 | 24 | 6 | 39 |
| 4 | 12 | 7 | 5 | 4 | 24 |
| 5 | 15 | 9 | 6 | 5 | 5 |
| 6 | 18 | 7 | 11 | 0 | 6 |
| 7 | 14 | 4 | 10 | 0 | 0 |
| 8 | 16 | 4 | 12 | 0 | 0 |
| 9 | 9 | 5 | 4 | 0 | 0 |
| 11 | 11 | 1 | 10 | 0 | 0 |
| Total | 201 | 87 | 114 | 57 | 230 |

# S5. Scenario and sensitivity analysis

**Model scenarios**

We examined several hypothetical scenarios in addition to the base case. This section describes the operationalization of these scenarios. The detailed results for the scenario analysis as described in Table 3 and Figure 6 in the main text.

**Base case (current TB control policy):** TB case finding in Songjiang relies heavily on passive detection, whereby individuals with TB-like symptoms report to community health centers and clinics [6]. There is at least one community health center in each sub-district. In addition, elderly residents can attend a free voluntary annual physical exam, which includes chest X-ray screening that can assist in identification of TB lesions.

**Scenario 1 (improved TB case finding in the elderly (65+ years-old)):** Residents aged >65 years currently receive an annual medical examination, which includes TB screening by chest X-ray, a process that has low sensitivity for active TB. This scenario assumes a 10% increase in sensitivity of this screening, through improvements in X-ray processing and interpretation.

**Scenario 2 (improved TB case finding and LTBI treatment in the elderly):** A version of Scenario 1 that adds LTBI screening and treatment for individuals testing negative for active TB.

**Scenario 3 (pre-employment entry TB screening for migrants):** A one-time TB screening for migrants at their time of first employment in Songjiang.

**Scenario 4 (pre-employment entry TB screening and LTBI treatment for migrants):** A version of Scenario 3 that adds LTBI screening and treatment for individuals testing negative for active TB.

**Scenario 5 (routine TB screening in the industrial zone and manufactories with high migrant workforce):** Regular active TB case-finding for in the manufacturing sector, which employs 62% of the migrant population (Figure S3). These manufactories mainly located in the industrial zone and some of them have their own staff dormitory and make it relatively feasible to set up targeted screening.

**Scenario 6 (all migrant-focused interventions):** The combination of interventions included in Scenarios 3-5.

**Scenario 7 (all interventions):** The combination of interventions included in Scenarios 1-5.

**Sensitivity analysis**

**Internal migration:** The estimated volume of internal migration from rural regions to cities and industrial zones is estimated to rise up to 240-260 million by 2030. Meanwhile, the megacities like Shanghai and Beijing have released government road map to stabilize the massive population migration into the city. We assumed an alternative 5% annual decease or increase of internal migration for sensitivity analysis (Figure S5 A-B).

**LTBI and TB prevalence:** As the overall improvement of national TB control effort, we tested the alternative assumption of 0% and 6% annual decline in LTBI and TB prevalence among the future entering migrants, compared to the 3% decline used in the main model. Figure S5 C-D shows the alternative assumption analysis.

**Long-time horizon projection:** we also assessed the main model outcomes based on the alternative base case with an extended 30-year time horizon (to 2050), as compared to 15 years (to 2035) used for the main analysis. (Figure S5 E and Table S7).

All of the alternative intervention in the scenarios (1-5) were assumed to be scaled up over 3 years, 2020-2022, using a piecewise linear function, then held in place for the rest of the simulation. Additional parameters for sensitivity analysis were summarized in Table 3 and Table S6.

## Table S6. Additional parameters used for model sensitivity analysis

| **Par_name** | **Mean value** | **Lower bound** | **Upper bound** | **Notes** |
| --- | --- | --- | --- | --- |
| *incr_sens* | 0.1 | 0.05 | 0.2 | Incremental improvement in sensitivity of TB screening among elderly residents |
| *tst_sens* | 0.88 | 0.8 | 0.95 | TST sensitivity [33] |
| *tltbi_init* | 0.35 | 0.2 | 0.5 | Prevent treatment uptake probability [34-37] |
| *tltbi_comp* | 0.7 | 0.6 | 0.8 | Prevent treatment completion probability [38] |
| *tltbi_cure* | 0.6 | 0.5 | 0.7 | Cure rate of prevent treatment [39] |
| *tbscr_sens* | 0.9 | 0.8 | 0.95 | TB screening sensitivity [40] |
| *rel_sens_early* | 0.5 | 0.25 | 0.75 | Relative rate of TB screening sensitivity for early status [40] |
| *tb_diag_sens* | 0.9 | 0.8 | 0.95 | TB diagnosis sensitivity based on symptom screen, smear test, liquid culture and GeneXpert MTB. |

* Parameters in this table are all assumed to be Beta distributed.

Figure S5. Sensitivity of the estimated change of TB notification and incidence with three assumption: different trends of migration volume—5% annual decease(A) or increase(B); decline of TB and latent *Mtb* infection prevalence (C, 0%, D, 6%) among future entering migrants; and an extended 30-year time horizon (estimation of outcomes until 2050, E). **** Table S7. Long-term horizon sensitivity analysis. Model estimated cumulated incremental scenarios of TB epidemiologic outcomes by migration status as a percentage of reduction compared to their base-case value by 2050*.

| Scenario | Transmissions | New *Mtb* infections | New TB cases | TB deaths | Year life lost to TB |
| --- | --- | --- | --- | --- | --- |
|  | Residents | | | | |
| Scenario 1 | 0.5 (0.2, 0.8) | 0.0 (0.0, 0.1) | 0.0 (0.0, 0.0) | 0.3 (0.1, 0.6) | 0.2 (0.1, 0.3) |
| Scenario 2 | 2.0 (1.2, 2.8) | 0.2 (0.1, 0.3) | 4.6 (2.6, 6.6) | 7.1 (4.2, 10.1) | 2.8 (1.6, 4.0) |
| Scenario 3 | 3.2 (1.8, 5.0) | 5.8 (3.3, 8.5) | 2.0 (1.1, 3.1) | 1.1 (0.6, 1.7) | 2.2 (1.2, 3.3) |
| Scenario 4 | 6.7 (4.4, 9.5) | 12.4 (8.6, 16.8) | 4.3 (2.7, 6.1) | 2.5 (1.5, 3.6) | 4.7 (3.0, 6.7) |
| Scenario 5 | 8.0 (4.7, 11.7) | 14.7 (9.2, 20.3) | 5.1 (3.0, 7.5) | 2.9 (1.7, 4.4) | 5.6 (3.2, 8.1) |
| Scenario 6 | 13.6 (8.9, 18.8) | 24.9 (17.2, 32.3) | 8.6 (5.5, 12.1) | 5.0 (3.2, 7.0) | 9.4 (6.1, 13.0) |
| Scenario 7 | 15.5 (10.8, 20.7) | 25.1 (17.4, 32.5) | 13.2 (9.4, 17.5) | 12.0 (8.5, 15.8) | 12.1 (8.6, 16.1) |
|  | Migrants | | | | |
| Scenario 1 | 0.0 (0.0, 0.0) | 0.0 (0.0, 0.0) | 0.0 (0.0, 0.0) | 0.0 (0.0, 0.0) | 0.0 (0.0, 0.0) |
| Scenario 2 | 0.0 (0.0, 0.0) | 0.0 (0.0, 0.1) | 0.0 (0.0, 0.0) | 0.0 (0.0, 0.0) | 0.0 (0.0, 0.0) |
| Scenario 3 | 6.2 (3.6, 9.0) | 6.4 (3.7, 9.4) | 2.7 (1.4, 4.1) | 2.8 (1.7, 4.1) | 3.4 (2.1, 4.9) |
| Scenario 4 | 13.1 (9.1, 17.8) | 13.2 (9.1, 18.1) | 9.6 (6.2, 13.5) | 9.3 (6.0, 13.0) | 9.9 (6.7, 13.7) |
| Scenario 5 | 15.7 (9.8, 21.5) | 15.9 (9.9, 21.8) | 6.6 (3.8, 9.7) | 7.7 (5.1, 10.6) | 8.9 (5.8, 12.1) |
| Scenario 6 | 26.5 (18.3, 34.0) | 26.7 (18.4, 34.5) | 15.2 (10.5, 20.1) | 15.9 (11.4, 20.5) | 17.5 (12.8, 22.4) |
| Scenario 7 | 26.5 (18.3, 34.0) | 26.8 (18.4, 34.5) | 15.2 (10.5, 20.1) | 15.9 (11.4, 20.6) | 17.5 (12.8, 22.4) |
|  | All | | | | |
| Scenario 1 | 0.1 (0.0, 0.1) | 0.0 (0.0, 0.0) | 0.0 (0.0, 0.0) | 0.2 (0.1, 0.4) | 0.1 (0.0, 0.1) |
| Scenario 2 | 0.3 (0.2, 0.5) | 0.1 (0.0, 0.1) | 1.6 (0.8, 2.5) | 3.6 (2.0, 5.4) | 0.9 (0.5, 1.4) |
| Scenario 3 | 5.7 (3.3, 8.4) | 6.2 (3.6, 9.2) | 2.4 (1.3, 3.8) | 2.0 (1.1, 3.0) | 3.0 (1.7, 4.5) |
| Scenario 4 | 12.2 (8.3, 16.7) | 13.0 (8.9, 17.8) | 7.8 (4.9, 11.0) | 5.8 (3.5, 8.5) | 8.2 (5.4, 11.6) |
| Scenario 5 | 14.6 (8.9, 20.1) | 15.6 (9.7, 21.5) | 6.1 (3.5, 9.0) | 5.3 (3.1, 7.7) | 7.8 (4.9, 11.0) |
| Scenario 6 | 24.6 (16.9, 32.2) | 26.3 (18.0, 34.1) | 13.0 (8.7, 17.6) | 10.3 (6.8, 14.3) | 14.9 (10.3, 19.5) |
| Scenario 7 | 24.9 (17.0, 32.4) | 26.4 (18.2, 34.1) | 14.5 (10.0, 19.2) | 13.9 (9.9, 18.4) | 15.8 (11.2, 20.4) |

* Color scale denotes magnitude of values (green higher, red lower), and is specific to each outcome (i.e., each column) and consistent through the population groups. Scenarios 1-2, resident-focused interventions; scenarios 3-5, migrant-focused interventions; scenarios 6, combined migrant-focused interventions; scenario 7, all combined interventions.
